# Prediction of prolonged mechanical ventilation in patients in the intensive care unit A cohort study

**Published:** 2013-09-30

**Authors:** Alvaro Sanabria, Ximena Gómez, Valentín Vega, Luis Carlos Domínguez, Camilo Osorio

**Affiliations:** 1 Head and neck service. Department of Surgery. Fundación Abood Shaio. Bogota. Colombia; 2 Department of Surgery. Universidad de La Sabana. Chía, Colombia.; 3 Universidad de Antioquia. Medellin, Colombia.

**Keywords:** tracheostomy, artificial respiration, intensive care units, ventilator weaning, intratracheal intubation, risk factors

## Abstract

**Introduction:**

There are no established guidelines for selecting patients for early tracheostomy. The aim was to determine the factors that could predict the possibility of intubation longer than 7 days in critically ill adult patients.

**Methods:**

This is cohort study made at a general intensive care unit. Patients who required at least 48 hours of mechanical ventilation were included. Data on the clinical and physiologic features were collected for every intubated patient on the third day. Uni- and multivariate statistical analyses were conducted to determine the variables associated with extubation.

**Results:**

163 (62%) were male, and the median age was 59±17 years. Almost one-third (36%) of patients required mechanical ventilation longer than 7 days. The variables strongly associated with prolonged mechanical ventilation were: age (HR 0.97 (95% CI 0.96-0.99); diagnosis of surgical emergency in a patient with a medical condition (HR 3.68 (95% CI 1.62-8.35), diagnosis of surgical condition-non emergency (HR 8.17 (95% CI 2.12-31.3); diagnosis of non-surgical-medical condition (HR 5.26 (95% CI 1.85-14.9); APACHE II (HR 0.91 (95% CI 0.85-0.97) and SAPS II score (HR 1.04 (95% CI 1.00-1.09) The area under ROC curve used for prediction was 0.52. 16% of patients were extubated after day 8 of intubation.

**Conclusions:**

It was not possible to predict early extubation in critically ill adult patients with invasive mechanical ventilation with common clinical scales used at the ICU. However, the probability of successfully weaning patients from mechanical ventilation without a tracheostomy is low after the eighth day of intubation.

## Abstract (es)

**Introducción:**

No existen guías establecidas para seleccionar los pacientes para traqueostomía precoz. El objetivo del estudio fue determinar los factores que pueden predecir la posibilidad de intubación mayor a 7 días en pacientes adultos en la UCI.

**Métodos:**

Este es un estudio de cohorte realizado en la UCI. Los pacientes en ventilación mecánica mayor a 48 horas fueron incluidos. Se recogieron los datos clínicos y fisiológicos de cada paciente al tercer día de intubación. Se realizaron análisis uní y multivariado para determinar las variables asociadas a intubación prolongada.

**Resultados:**

163 (62%) fueron hombres y la edad media fue de 59±17 años. Aproximadamente un tercio de los pacientes (36%) requirieron ventilación mecánica mayor a 7 días. Las variables asociadas con ventilación mecánica prolongada fueron: edad (HR 0.97 (95% CI 0.96-0.99); diagnóstico de urgencia quirúrgica en un paciente con enfermedad médica (HR 3.68 (95% CI 1.62-8.35),diagnostico de necesidad de cirugía no urgente (HR 8.17 (95% CI 2.12-31.3); diagnóstico de enfermedad medica no quirúrgica (HR 5.26 (95% CI 1.85-14.9); APACHE II (HR 0.91 (95% CI 0.85-0.97) y puntaje SAPS II (HR 1.04 (95% CI 1.00-1.09). El área bajo la curva ROC para predicción fue de 0.52. 16% de los pacientes fueron extubados después de 8 días de intubación.

**Conclusiones:**

No fue posible predecir la extubation precoz en pacientes de la unidad de cuidado intensivo en ventilación mecánica con las variables comúnmente usadas. La probabilidad de destete de ventilación mecánica después de 8 días de intubación sin realizar traqueostomía es baja.

## Introduction

Tracheostomy is the most common elective surgical procedure performed by surgeons in intensive care patients. Almost up to 24% of patients in the intensive care unit (ICU) need a tracheostomy^1^ ^.^Tracheostomy has been recommended because its potential advantages over tracheal intubation as there is a lower frequency of laryngeal ulcers, less resistance in the airway, less dead space and better tolerance by patients^2^. Currently, the main indications for tracheostomy are prolonged mechanical ventilation (because of the possibility of reduced mechanical ventilation time), length of stay in the ICU and associated mortality^3^.

However, the prediction of prolonged mechanical ventilation is challenging. Currently, there is not a validated instrument that predicts which patients in the ICU will require prolonged ventilation and are candidates to early tracheostomy. Most decisions are based on clinical criteria, which are often late, subjective and unreliable^4^

Some randomized controlled trials have shown that an early tracheostomy^2^ (made before the seventh day of tracheal intubation) has benefits for the patient, but the challenge is determining which patients will be eligible for an early surgical procedure. The objective of this study was to identify and assess the factors that could help to predict prolonged mechanical ventilation defined as longer than 7 days, considering this time the limit to define an early or late tracheostomy, in patients during the first three days of their ICU stay and could be used in the clinical decision-making. We consider three days is an enough time to control major clinical conditions and to define the next steps about mechanical ventilation plan, including extubation or long term maintenance of mechanical ventilation.

## Methods

This study was a cohort study and approved by the Ethics in Research Committee. We reviewed the medical charts of adult patients who were admitted to a general ICU at the Fundacion Abood Shaio Clinic, Bogota, Colombia, between January 1, 2007, and December 31, 2008, that required at least 48 hours of mechanical ventilation with tracheal intubation. The decision to perform a tracheostomy or to wean from mechanical ventilation was made exclusively by the ICU physician and was based on their clinical judgment at the time of the extubation, considering the classical hemodynamic and pulmonary factors, sepsis control, etc. 

Patients with neck conditions and non-invasive ventilation who were intubated at an external institution for acute airway compression, patients transferred to other ICUs and patients with a survival less than seven days after intubation were excluded to reduce the risk of selection bias. Data were collected from the medical records by an independent reviewer using a specifically designed form at the third day of intubation. The form included demographic (age, sex, weight, height), clinical (diagnosis prior to ICU admission, indications for mechanical ventilation, SOFA score, APACHE II score, SAPS II score, Glasgow Coma Score, vital signs, laboratory and imaging data) and therapeutic (vasoactive drugs, antibiotics, immunosuppressants and bronchodilators) variables, commonly used at ICU. The main outcome was time to extubation, measured in days. The scores selected (SOFA, APACHE II and SAPS II) were used as variables to adjust for the final outcome, in order to avoid selection bias. 

A sample size calculation of 350 patients was made using the Carley nomogram, assuming that classical variables were the predictors of mechanical ventilation discontinuation. We selected a rate of prolonged mechanical ventilation (longer than 7 days) of 20%, a sensitivity of 90%, and a specificity of 90% and a precision of 7% for any prediction variable. The information from the forms was entered into a database (Excel 2007, Microsoft Corp, ).

For statistical analysis, commercially available software was used (Stata 9.0, Stata Corporation, College Station, Texas). Descriptive statistics were used to show the distribution of variables (mean ± sd, median and range for continuous variables and frequency for discrete variables). As mechanical ventilation was a time-to-event outcome, we calculated the time from the date of the intubation until the thirtieth day after to admission. A univariate analysis using the Kaplan-Meier method was used to explore the relationship between the baseline variables and outcome events, and the results were reported using a Mantel-Cox hazard ratio (HR) with a 95% CI. The log-rank test was used to define the statistical significance. A Cox proportional hazard regression analysis was used to assess the independent effect of the variables on mechanical ventilation. For ordinal variables, an indicator variable was created for each level in the logistic and Cox proportional hazard regression analysis. As many clinical variables are used by different scores, we decided to include only scores in the regression analysis in order to avoid collinearity. The predictive capability of the model was assessed with an area under ROC curve analysis. For all statistical tests, *p*<0.05 was considered statistically significant.

## Results

**Figure 1:**
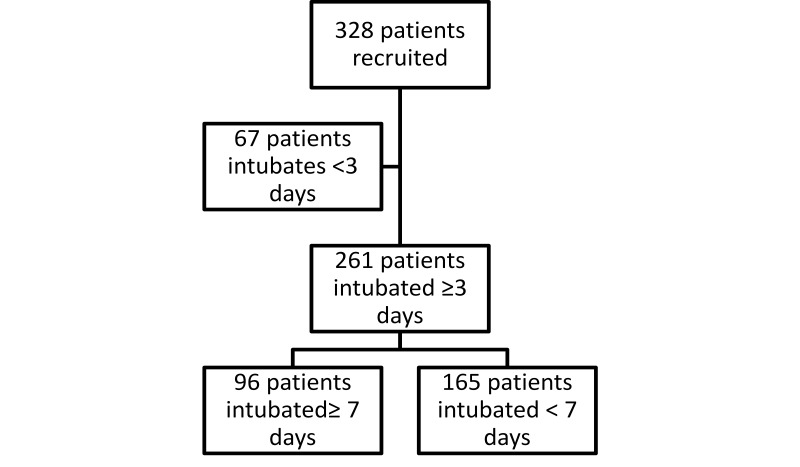
Flow diagram

The clinical charts of 328 patients fulfilled the inclusion and exclusion criteria. From these, 261 patients were still intubated on the third day, which was the population studied. Figure 1. The mean age was 59.6 ± 17.8 years (range 18-97; median 64 years), and 163 patients were men (62.4%). Table 1. The admission diagnoses to the ICU are shown in Table 2. They were classified as surgical or non-surgical and emergent or non-emergent, and the presence of medical, surgical or trauma conditions were noted. The indications for ventilatory support were: surgery in 115 patients (44.1%), hemodynamic instability in 19 patients (7.3%), respiratory failure in 70 patients (26.8%) and Glasgow coma score <8 in 57 patients (21.8%) 

**Table 1:**
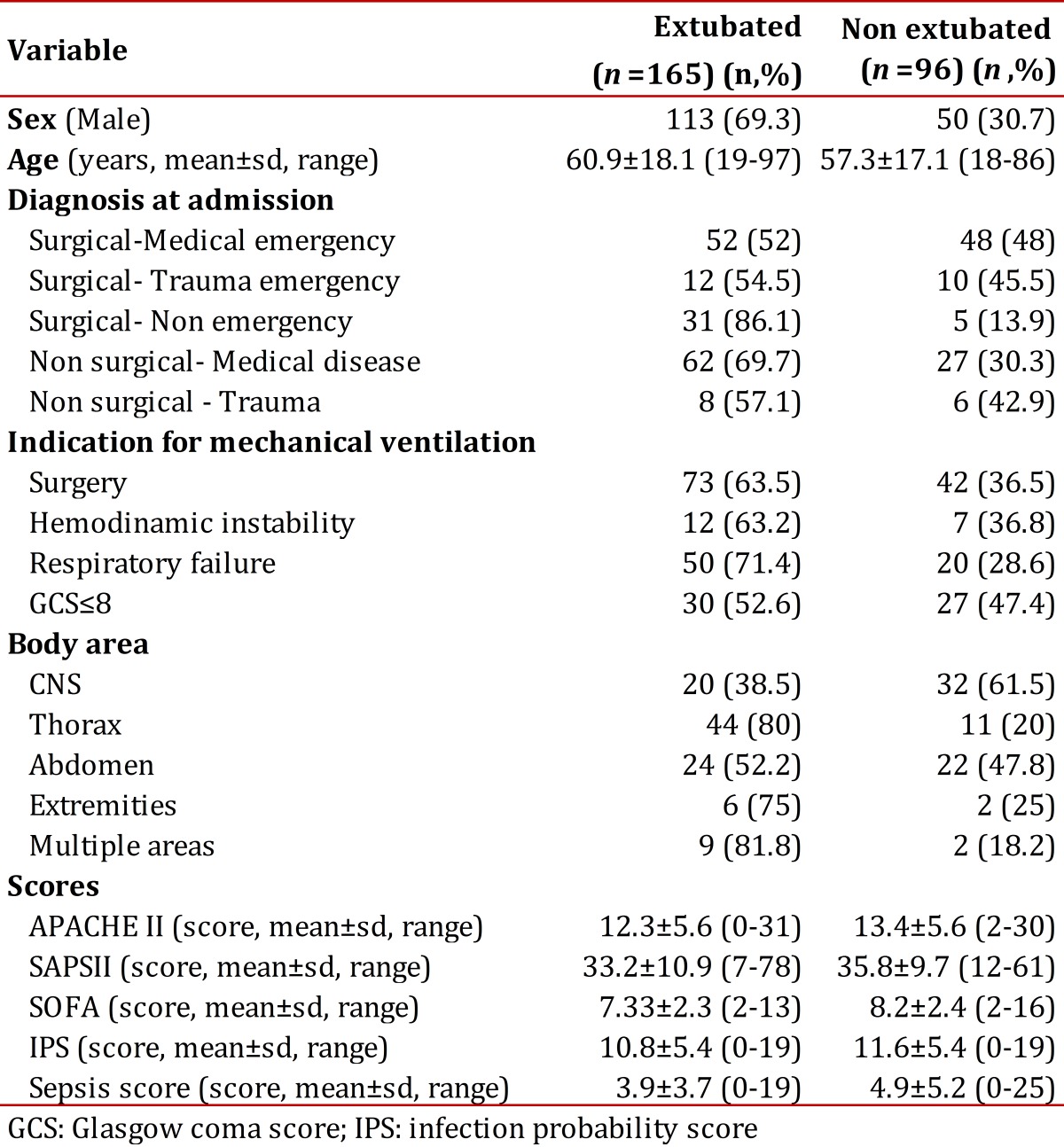
Characteristics of patients according to intubation status.

**Table 2:**
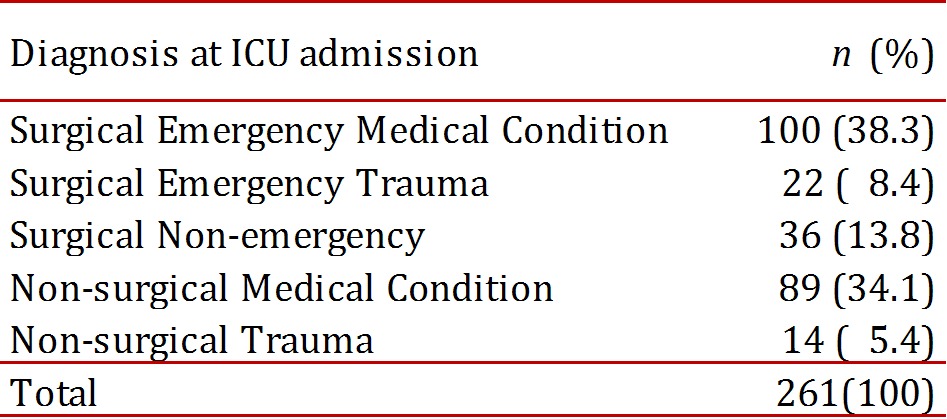
Distribution according to the diagnosis at admission to the ICU

**Figure 2.**
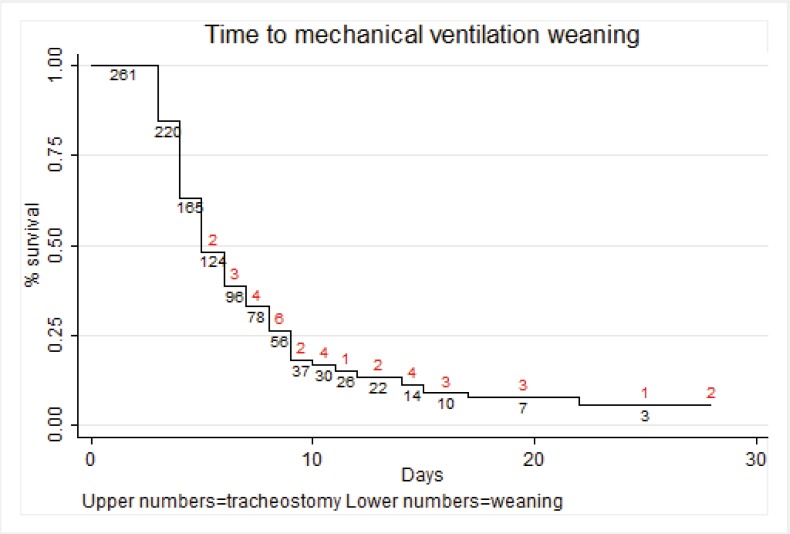
Kaplan-Meier graph for extubation.

The mean score and standard deviation at admission for SOFA was 7.9 ± 2.3 (range 2-15), for APACHE II was 15.1 ± 5.7 (4-31), for SAPS was 37.9 ± 10.6 (9-87) and for GCS was 12.3 ±3.7 (3-15). The mean time of mechanical ventilation was 6.6± 4.2 days (3-28, median 5 days). Ninety-six patients (36.8%) were intubated more than 6 days, and 37 patients (14.2%) underwent tracheostomy at a mean time of 9.5± 4 days (4-20), 75.7% of them after day 7. Figure 2 shows a Kaplan-Meier graph with the weaning of mechanical ventilation as the main outcome and tracheostomy as lost events. The univariate analysis results are shown in table 3.

**Table 3:**
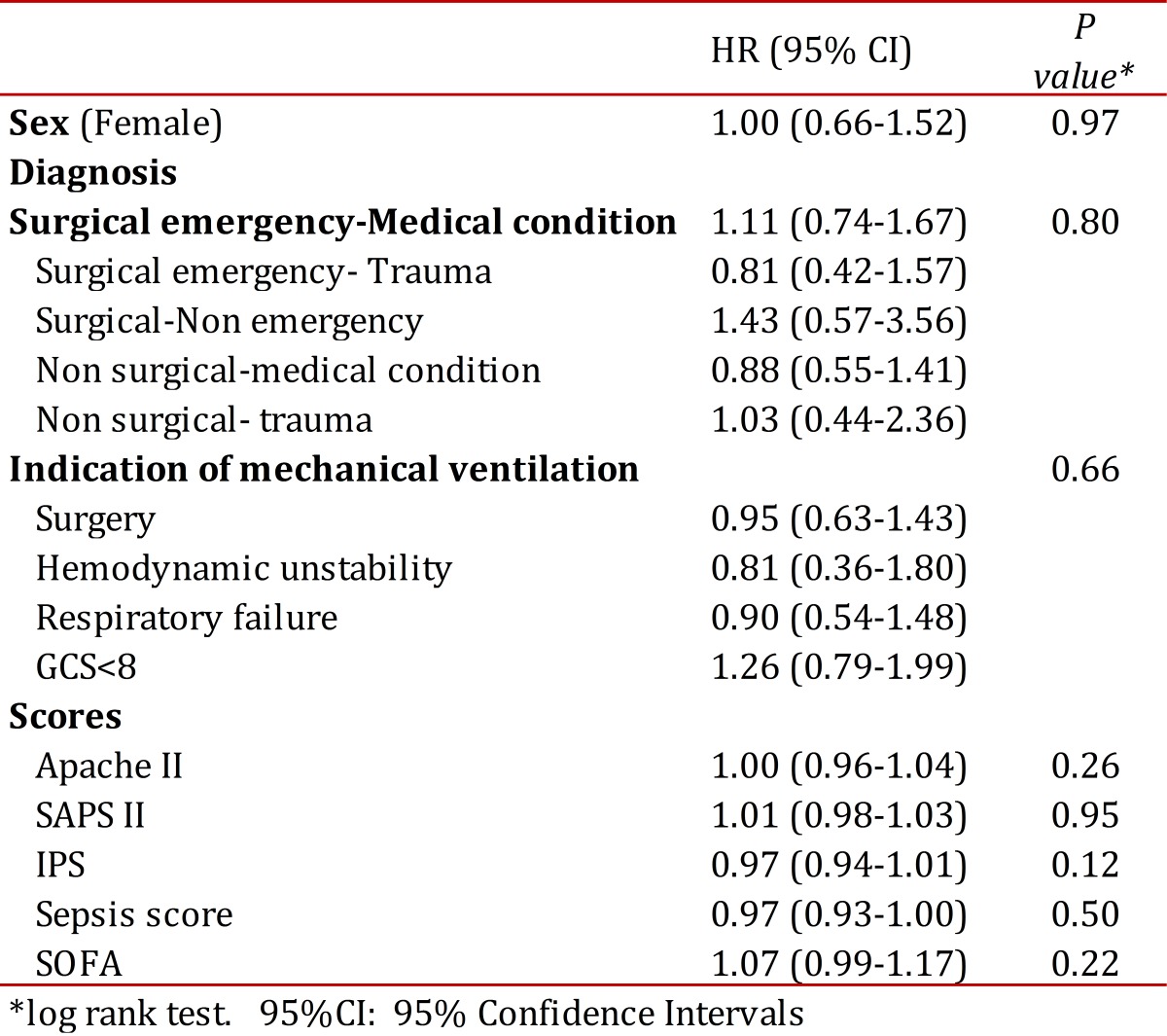
Uniariate analysis for intubation >7 days

The multivariable Cox model included gender, age, diagnosis at admission, indication of mechanical ventilation, APACHE II, SAPS and SOFA. The final model contained the following clinically and statistically significant variables: age (HR 0.97 (95% CI 0.96-0.99); diagnosis of surgical emergency in a patient with a medical condition (HR 3.68 (95% CI 1.62-8.35),diagnosis of surgical condition-non emergency (HR 8.17 (95% CI 2.12-31.3); diagnosis of non surgical-medical condition (HR 5.26 (95% CI 1.85-14.9); APACHE II (HR 0.91 (95% CI 0.85-0.97) and SAPS II (HR 1.04 (95% CI 1.00-1.09) . The prediction based on these variables had an area under ROC curve of 0.52, which is considered poor.

The life table derived from the Kaplan-Meier analysis provides clinicians with data that could assist in decision-making for tracheostomy. At day 8 and 10, the probability of being extubated was 25.7% and 16.2%, respectively. 

## Discussion

Tracheostomy has been performed since 1500 BC^5^.Today, tracheostomy is performed in nearly 25% of all patients admitted to the ICU and is commonly indicated for prolonged mechanical ventilation^1^ ^,^ ^6^ because it is associated with less larynx and oral ulcers, less ventilatory resistance and dead space, increased comfort and secretions management, less sedative requirements and ease of communication and oral feeding^7^.Currently, some studies have shown that there are significant benefits of early tracheostomy (less than 7 days of intubation) in intubated patients. In 1989, the American College of Chest Physicians published a consensus document that recommended early tracheostomy in patients with an estimated intubation time longer than 21 days^8^.Later, Brook *et al* ^9^. found that patients with early tracheostomy had lower hospital costs in a prospective trial with 90 patients, and Hsu et al^1^. showed that patients who underwent to tracheostomy after three weeks of mechanical ventilation had a higher frequency of re-intubation and increased mortality. A meta-analysis conducted in 2005 reported a decreased length of stay in the ICU (15.3 days (CI 95%: 6.1 - 24.6)) and less time on mechanical ventilation (8.5 days (CI 95%: 1.7- 15.3)),^2^ and Freeman et al^10^. confirmed these results in an administrative data study. Therefore, patients with a predicted prolonged intubation would benefit from a tracheostomy made within the first week of intubation. Recent studies from Germany^11^ ^,^ ^12^, USA^13^, Spain^14^ and Italy^15^, have shown an improvement in secondary outcomes as length of stay at the ICU, days of mechanical ventilation, ventilator associated pneumonia but not in mortality.

However, it is difficult to predict prolonged intubation. In 1990, Heffner et al^4^. found that a PEEP less than or equal to 10 cm H2O, a PaO2/PAO2 ratio higher than 0.4 and a quick improvement in radiologic findings in a thorax x-ray were associated with a mechanical ventilation time less than 14 days. Troche et al^16^. showed that the serum albumin, indications for intubation (respiratory failure), sepsis scale score, number of organs in failure and lung injury scale score were predictors of prolonged mechanical ventilation. However, the validation of these factors had a low predictive value (24%). Sellers et al^17^.found that age and a PaO2/FiO2 on the third day of intubation were predictors of prolonged intubation. On the other hand, Kollef et al^18^.found that nosocomial pneumonia, aerosolized medication administration, re-intubation and lung aspiration increased the necessity for tracheostomy. Other authors have reported that bilateral blunt thoracic trauma, age, brain trauma, previous diagnosis of COPD, high APACHE-II score, high SAPS scores, high Glasgow Coma Scores, BMI>20 Kg/m2, levels of CRP>10 mg/L are also predictors of prolonged ventilation^19^ ^,^ ^20^

Although many predictive factors have been identified, research has been very heterogeneous related to the study populations (surgical, trauma) such that the results cannot be extrapolated to other populations; a different scale is required for each type of patient. Currently, there is not a simple scale that predicts time of mechanical ventilation in patients admitted to a general ICU, and therefore, the indications for an early tracheostomy remain subjective. 

This study demonstrated that the factors analyzed that have shown good reliability for other outcomes and which are currently used at ICU around the world, are poor predictors of prolonged mechanical ventilation. Furthermore, the predictive power of the factors identified in the multivariate analysis are so poor that it is impossible to predict prolonged intubation. 

This finding can be explained by the dynamic nature of the ICU patients that could have significant clinical changes in a few hours, making the initial factors irrelevant as time passes. On the other hand, the variability of treatments and clinical criteria between physicians can obscure the identification of predictive factors, and the interaction between the known and unknown factors could hide the real predictive factors.

However, this study also offers information on the time of tracheostomy based using a survival analysis. As shown, the probability for extubation is approximately 25% after day 8 and less than 18% after day 9. Despite the variability, it is reasonable to suggest that patients who reach this day are good candidates for tracheostomy based on their low realistic probability of being extubated. Furthermore, if patients who have been intubated for six days are likely to be extubated within the next two days, physicians may simply choose to observe the patients; on the contrary, if the estimated time of mechanical ventilation is longer, they can perform a tracheostomy earlier. 

Finally, some weaknesses of this study should be addressed. First, the observational design is intrinsically prone to bias, and second, the variability of decisions by physicians impedes the use of standards and reliable criteria for uniform decision-making. Although we tried to control for these weaknesses using objective criteria, they could affect the final results and should be analyzed carefully. 

In conclusion, it was not possible to identify the predictive factors for prolonged mechanical ventilation, and decisions for tracheostomy cannot be based on these factors. However, an intubation time longer than 8 days is associated with a low probability of extubation and can be used as a time point in the decision to perform a tracheostomy. 
